# Biomechanical comparison between single-bundle and double-bundle anterior cruciate ligament reconstruction with hamstring tendon under cyclic loading condition

**DOI:** 10.1186/1758-2555-4-23

**Published:** 2012-07-02

**Authors:** Shuya Nohmi, Yasuyuki Ishibashi, Eiichi Tsuda, Yuji Yamamoto, Harehiko Tsukada, Satoshi Toh

**Affiliations:** 1 Department of Orthopaedic Surgery, Hirosaki University Graduate School of Medicine, 5 Zaifu-cho, Hirosaki, Aomori, 036-8562, Japan

## Abstract

**Purpose:**

The purpose of this study was to compare the anterior tibial translation (ATT) of the anterior cruciate ligament (ACL) reconstructed-knee between single-bundle and double-bundle ACL reconstruction under cyclic loading.

**Methods:**

Single-bundle and double-bundle reconstructions of the knee were performed sequentially in randomized order on the same side using eight human amputated knees. After each reconstruction, the reconstructed-knee was subjected to 500-cycles of 0 to 100-N anterior tibial loads using a material testing machine. The ATT before and after cyclic loading and “laxity increase”, which indicated a permanent elongation of the graft construct, was also determined.

**Results:**

The ATT after cyclic loading increased in both single-bundle and double-bundle reconstruction techniques compared to that without cyclic loading. Changes in ATT before and after cyclic loading were 3.9 ± 0.9 mm and 2.9 ± 0.6 mm respectively, and were significantly different. Laxity increase was also significantly different (4.3 ± 0.9 mm and 3.2 ± 0.8 mm respectively). Although no graft rupture or graft fixation failure was found during cyclic loading, the graft deviated into an eccentric position within the tunnel.

**Conclusions:**

Although ATT was significantly increased in both single-bundle and double-bundle reconstruction with hamstring tendon after cyclic loading test, there was significant difference. Double-bundle reconstruction might be superior to prevent increasing ATT under cyclic loading. Deformation of hamstring tendon after cyclic loading might result in deterioration of knee stability after ACL reconstruction, and is one of disadvantages of soft tissue graft.

## Introduction

The anterior cruciate ligament (ACL) is a commonly injured ligament within the knee joint, and arthroscopic single-bundle (SB) ACL reconstructions have been performed worldwide using biological substitutes, such as bone-patella tendon- bone autograft, hamstring tendon autograft, and allograft. Although most patients have their normal knee function restored and can return to their pre-injury activity level after SB reconstruction, some patients still feel instability even though their reconstructed knee is stable as measured by Lachman test and an instrumented knee laxity device such as the KT-1000 arthrometer [1]. This means that traditional SB reconstruction techniques do not completely reproduce the original ACL [2-4], which anatomically and functionally consists of two bundles: the anteromedial (AM) bundle and the posterolateral (PL) bundle [5]. Therefore, double-bundle (DB) ACL reconstructions, which reproduce both the AM and PL bundles, have been developed to improve clinical outcomes. Although several recent Level I and II studies have shown that DB reconstruction had superior short-term results than SB reconstruction [6-10], some studies showed no advantage for DB reconstruction [11], so it is still controversial.

Biomechanical advantages of DB reconstruction were first demonstrated by Radford and Amis [12] using a materials testing machine (Instron 1122), which could only apply an anterior-posterior (AP) load. Recently, new robotic technologies have been introduced into the knee biomechanical studies [13]. This technology can apply more complex loads, such as varus/valgus and rotational loads, and has demonstrated the functional role of the AM and PL bundle as well as biomechanical advantages of anatomical DB reconstruction, especially to manipulate a combined rotatory load that mimics the pivot shift test [4,14-16]. Although these robotic studies demonstrate the complex function of the ACL, only a single testing condition or a small number of loads were used. In actuality, implanted graft materials within the knee joint must bear cyclic loading during daily activities and also in postoperative rehabilitation [17-21]. There have been few studies which compare SB and anatomical DB ACL reconstruction under cyclic loading conditions. Therefore, the purpose of this study was to compare the anterior tibial translation (ATT) of the ACL reconstructed-knee under cyclic loading between SB and DB ACL reconstruction. It was hypothesized that the DB reconstruction would decrease ATT compared to the SB technique, and would subsequently reduce ATT increase during cyclic loading.

## Materials and methods

**Figure 1:**
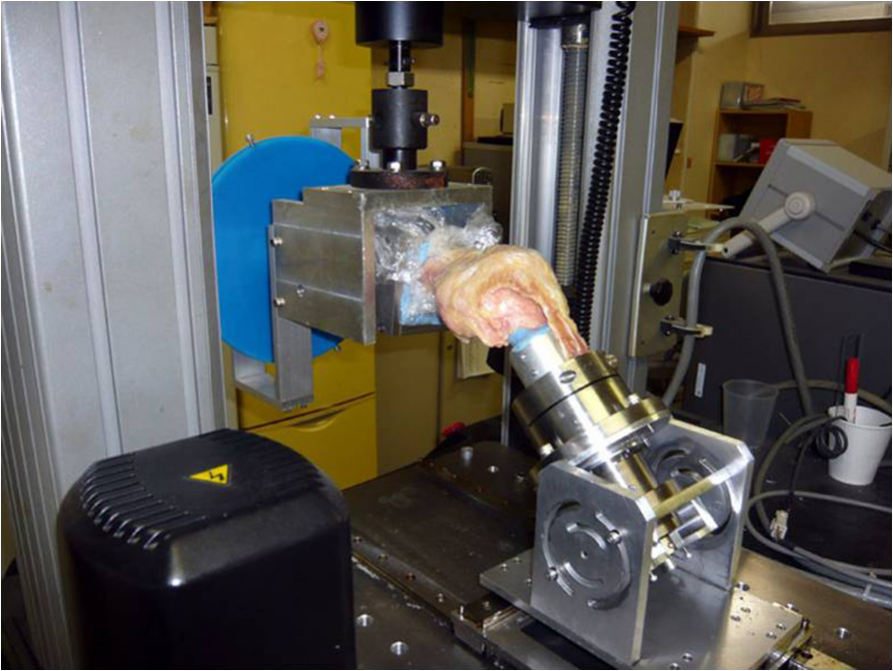
Photograph of the testing system with knee joint at 30° of flexion.

Eight fresh-frozen amputated human knees with an average age of 70.6 (18–93) years were used in this study. Specimens had no prior surgery or evidence of abnormal laxity, and obvious degenerative knees were excluded. Informed consent was obtained from the patients and ethical approval of this study was obtained from the Ethics Committee of Hirosaki University School of Medicine. Specimens stored at −80°C were thawed at room temperature 24 hours before testing and kept moist with saline spray during preparations and mechanical testing [22]. Surrounding skin and muscles were removed to expose the bone, leaving the knee ligaments and popliteus muscle and tendon intact. The extensor mechanism was removed because it has no significant effect on anterior-posterior (A-P) drawer motion up to 90° of flexion [23]. The proximal part of the femur and the distal part of the tibia were placed in custom-made metallic pots of polymethylmethacrylate for gripping to test the fixture’s rigidity. The specimens were mounted on a materials testing machine (Instron 4465; Instron Corp, Canton, MA) with custom-made apparatus without restricting five degrees of freedom except for varus-valgus rotation (Figure 1).

### Testing protocol

**Figure 2:**
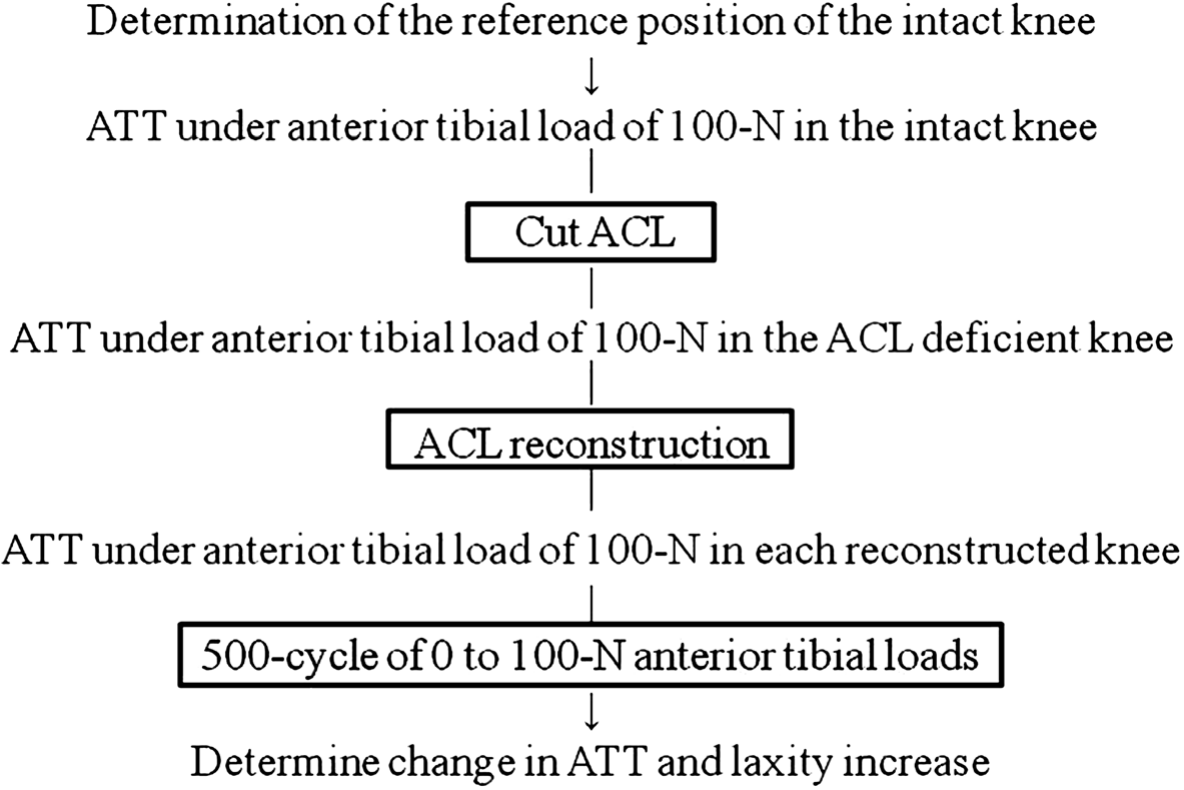
**Testing protocol and data obtained.** A-P: Anterior-posterior, ATT: Anterior tibial translation from neutral A-P position.

**Figure 3:**
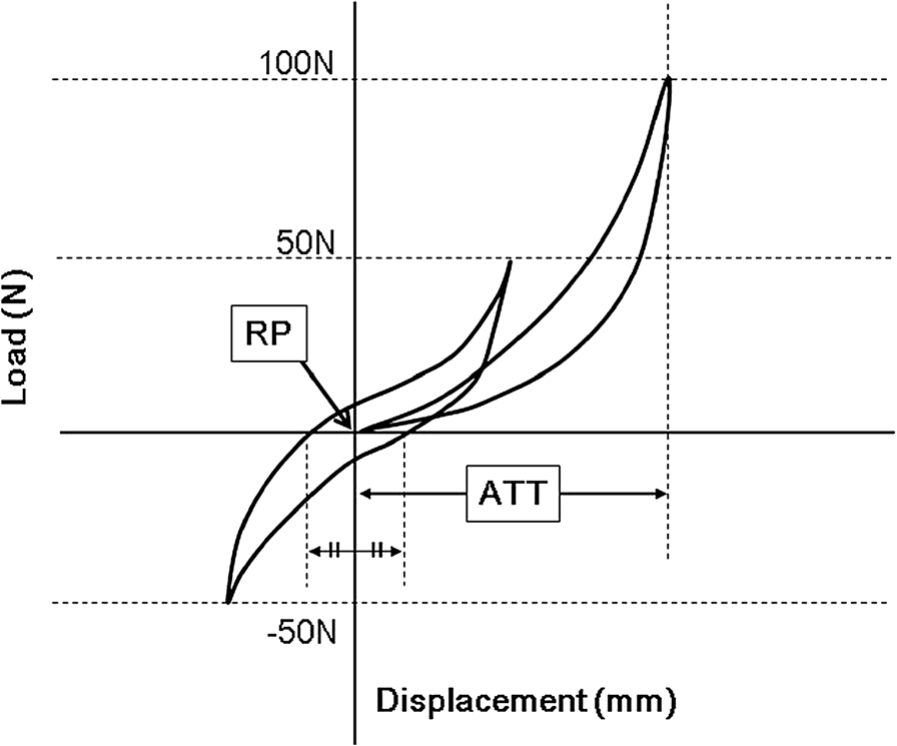
**Schematic of how to determine the reference position (RP) and the anterior translation of the tibia (ATT).** RP was defined as the position midway between the 2 zero points of the load–displacement hysteresis loop. ATT was defined as the displacement of the tibia from the RP with 100-N anterior tibial loads.

Before sectioning the ACL, the neutral anterior-posterior (A-P) position of the intact knee at 30° of knee flexion was determined and used as a reference position (RP) throughout the testing (Figure 2). It was defined as the position midway between the 2 zero points of the load–displacement hysteresis loop by imposing a ± 50-N drawer cycle [24] resulting in four degrees of freedom (DOF) knee kinematics (medial-lateral, and proximal-distal translations) (Figure 3). After determining the RP (A-P neutral position), the intact knee was subjected to 30 cycles of 0 to 100-N anterior tibial loads at a crosshead speed of 100 mm/min and load–displacement curve was recorded. Displacement of the tibia at 100-N anterior tibial loads from RP was defined as ATT (anterior translation of the tibia), and ATT of the intact knee (after 30 cyclic load) was recorded. Next, the ACL was transected to simulate an isolated ACL tear, and the same cyclic loading conditions were also applied to the ACL-deficient knee and the load–displacement curve was recorded.

**Figure 4:**
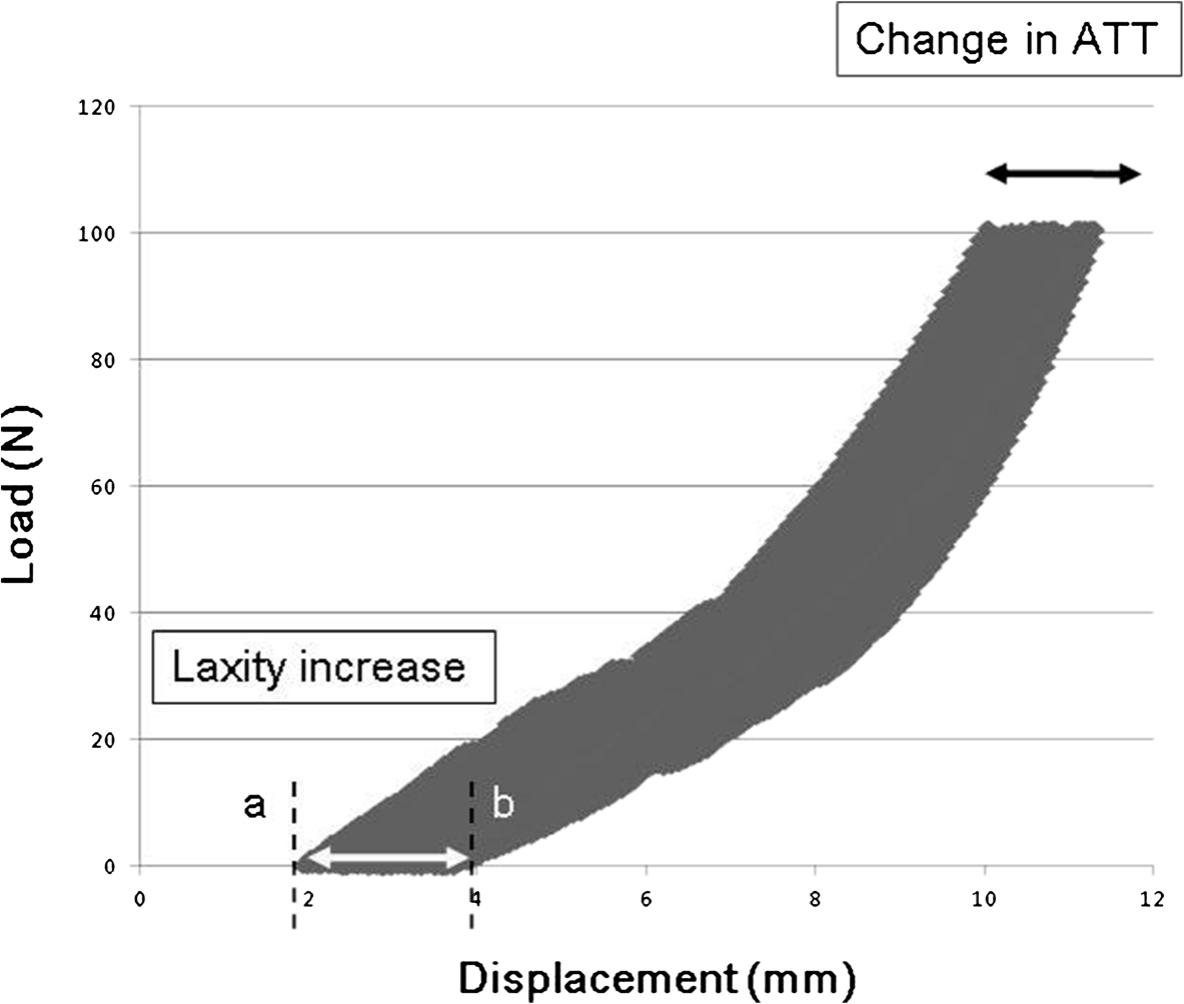
**Schematic of change in anterior tibial translation (ATT) and laxity increase in load–displacement curve during cyclic loading.** The laxity increase is distance between initial tibial position before cyclic loading and the final position after cyclic loading.

SB and DB-ACL reconstructions were performed sequentially in randomized order in the same knee. Each reconstructed knee was subjected to 500-cycles of 0 to 100-N anterior tibial loads at a crosshead speed of 100 mm/min and load–displacement curve was recorded. The ATT in response to an anterior load of 100-N before and after cyclic loading was determined in reference to the neutral A-P position. Additionally, the parameter “laxity increase” introduced by Scheffler et al. [24]. to quantify the loss of graft fixation was calculated as the change in the tibial position at load pickup between the first and last cycle of cyclic loading (Figure 4). The laxity increase is distance between initial tibial position before cyclic loading and the final position after cyclic loading. This causes a permanent elongation of the graft constructs, which consists of the graft slippage from the fixation device and plastic deformation of the linkage materials and knot tightening. Therefore, the ATT consists of the laxity increase and the recoverable elongation of the tendon graft itself.

### Surgical procedure

ACL graft materials were semitendinosus and/or gracilis tendons, which were harvested from each knee specimen during preparation and also other amputated human knees, which were used for another study. These tendons were trimmed, and cut in half if the tendon was long enough (over 24 cm). Both ends of each graft were connected with No. 2 Ethibond sutures (Ethicon, Inc., Somerville, NJ, US) using baseball glove sutures, and folded once (single loop). To decrease difference between SB- and DB-ACL reconstructions, each procedure used two looped graft (4 strands tendon) totally. However, graft conditions were not the same because grafts were used for each cyclic loading test. For SB-ACL reconstruction, double looped grafts were combined and used if the diameter was to be more than 6 mm (6-8 mm). For DB-ACL reconstruction, single looped grafts were used for the AM bundle and PL bundle graft, and diameters of grafts were usually about 5 mm (4.5-6 mm). These grafts were pretensioned with 20-lb during tunnel preparation using the Graft Master II (Smith & Nephew, Andover, MA, USA). A suture plate (B/Braun AESCULAP, Tuttlingen, Germany) was attached to the proximal end of each graft using No.5 Ethibond, and the length of the suture loop was matched to the femoral tunnel measured during reconstruction.

After cyclic loading test of intact and ACL-deficient knee, each specimen was detached once from the clamp. The ACL remnant was removed and both femoral and tibial footprints of the AM bundle and the PL bundle were carefully identified under direct visualization. Notch plasty was not performed. Then, SB- and DB-ACL reconstructions were performed in randomized order. After the first reconstruction and its testing, the created tunnels were packed by cancellous bone, and then the next reconstruction was performed. These cancellous bones were harvested from another specimen’s knee condyle using a coring reamer. Diameters of these coring reamers were 1 mm larger than the created tunnel. Therefore, the prior tunnel was firmly packed with solid bone to decrease adverse effects on the next reconstruction.

For SB-ACL reconstruction, the femoral and the tibial guide pins were inserted into the center of each footprint (middle point between the AM and PL bundle footprints). These pins were overdrilled outside-in by a cannulated drill matched with graft diameter. For DB-ACL reconstruction, the AM and PL guide pins were inserted into the center of the AM and PL bundle footprints with careful attention not to overlap the tunnels. These pins were overdrilled in the same manner as the SB-ACL reconstruction. Grafts for SB- or DB-ACL reconstructions were inserted from proximal (femoral tunnel) to distal (tibial tunnel) antegradely, and then the distal end of each graft was connected with a Double Spiked Plate (DSP; Meira Corp, Nagoya, Aichi, Japan) through No.2 Ethibond [25]. Knees were kept at 30° of knee flexion on the materials testing machine at the neutral position, and grafts were tensioned using a calibrated spring scale and fixed at 20-lb for SB-ACL reconstruction and 10-lb each (total of 20-lb) in DB-ACL reconstructions through the Double Spiked Plate.

### Statistical analysis

Statistical analyses were performed to compare ATT and laxity increase between SB-ACL reconstructed and DB-ACL reconstructed knees. For these biomechanical data, a paired t test was used. Statistical analyses were also performed to compare the ATT between intact knees, ACL deficient knees, SB-ACL reconstructed and DB-ACL reconstructed knees. For these biomechanical data, statistical evaluation was made using a two-way analysis of variance with the Tukey HSD test for post hoc multiple comparisons. The level of significance was set at P < 0.05. SPSS version 12.0 software (SPSS Inc., Chicago, Illinois, USA) was used for statistical analysis in this study.

## Results

**Figure 5:**
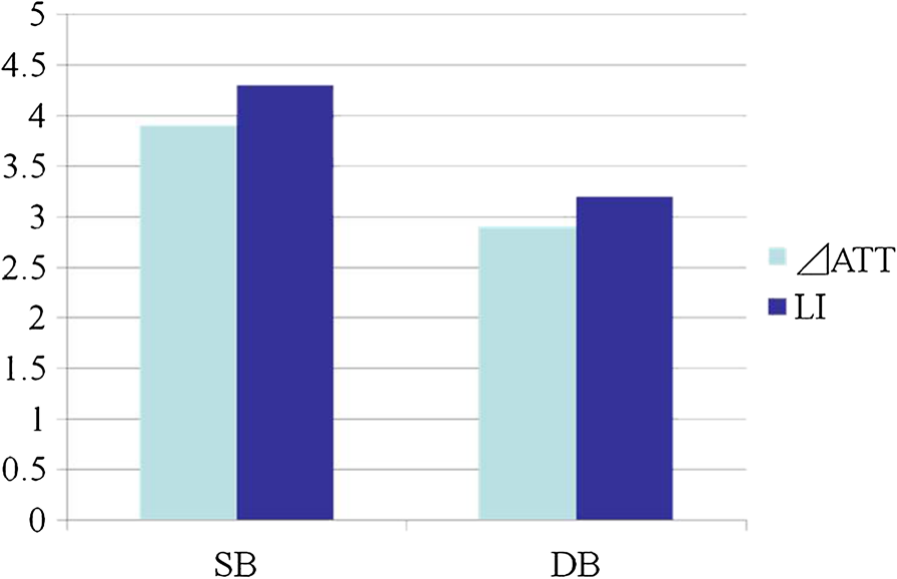
**Change in anterior tibial translation (**Δ**ATT)and “laxity increase” (LI) of ACL reconstructed-knees during cyclic loading.** Those in SB reconstruction were significantly greater compared to the DB reconstruction.

ATT in the intact knee (9.1 ± 3.9 mm) was significantly increased in the ACL-deficient knee (16.7 ± 4.3 mm) (P = 0.003) (Table 1). Before cyclic loading, ATT in the ACL-deficient knee was significantly improved in both SB (11.8 ± 3.6 mm) and DB reconstruction (10.1 ± 4.0 mm), and there were no significant differences between the reconstruction techniques (P = 0.961). ATT after cyclic loading increased in both reconstruction techniques compared to that without cyclic loading. ATT after cyclic loading was 15.7 ± 4.1 mm in SB reconstruction (P = 0.077, compared with before cyclic loading) and 13.1 ± 3.9 mm in DB reconstruction (P = 0.677). There was significant difference in the ATT after cyclic loading between SB and DB reconstruction (P = 0. 027). Therefore, changes in ATT between before and after cyclic loading were also significantly different (3.9 ± 0.9 mm and 2.9 ± 0.6 mm, respectively) (Figure 5). Laxity increases in SB and DB reconstruction were 4.3 ± 0.9 mm and 3.2 ± 0.8 mm respectively, and there was also significant difference (P = 0.021).

**Table 1:** Anterior tibial translation and laxity increase during cyclic loading

**Anterior tibial translation (ATT)**							
	Before cyclic loading		After cyclic loading		ΔATT	Laxity increase	
Intact knee	9.1 ± 3.9 mm						
ACL deficient	16.7 ± 4.3 mm						
SB-ACLR	11.8 ± 3.6 mm		15.7 ± 4.1 mm		3.9 ± 0.9 mm	4.3 ± 0.9 mm	
		P = 0.961		P = 0. 027			P = 0.021
DB-ACLR	10.1 ± 4.0 mm		13.1 ± 3.9 mm		2.9 ± 0.6 mm	3.2 ± 0.8 mm	

SB-ACLR: single –bundle ACL reconstruction, DB-ACLR: double –bundle ACL reconstruction, ΔATT: change in anterior tibial translation.

**Figure 6:**
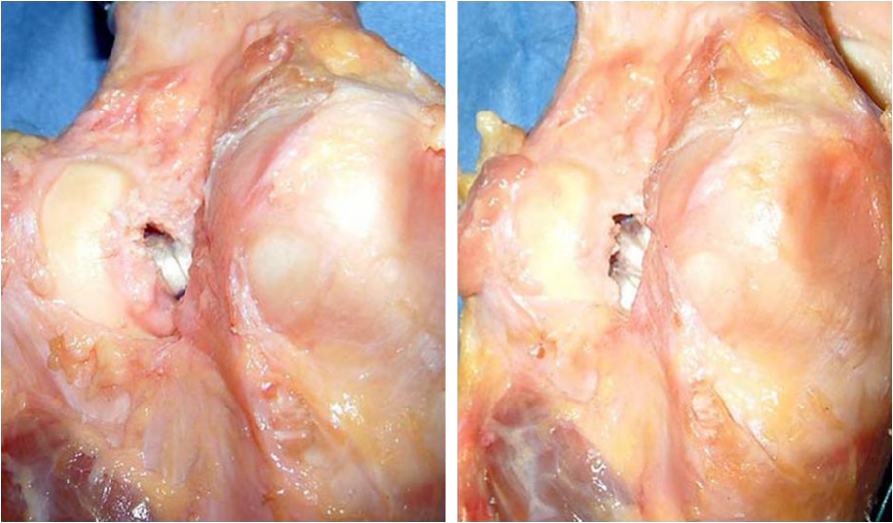
**Eccentric graft positioning within femoral tunnels in single-bundle (left) and double-bundle reconstruction (right).** Posterior capsule was removed to show graft positioning

**Figure 7:**
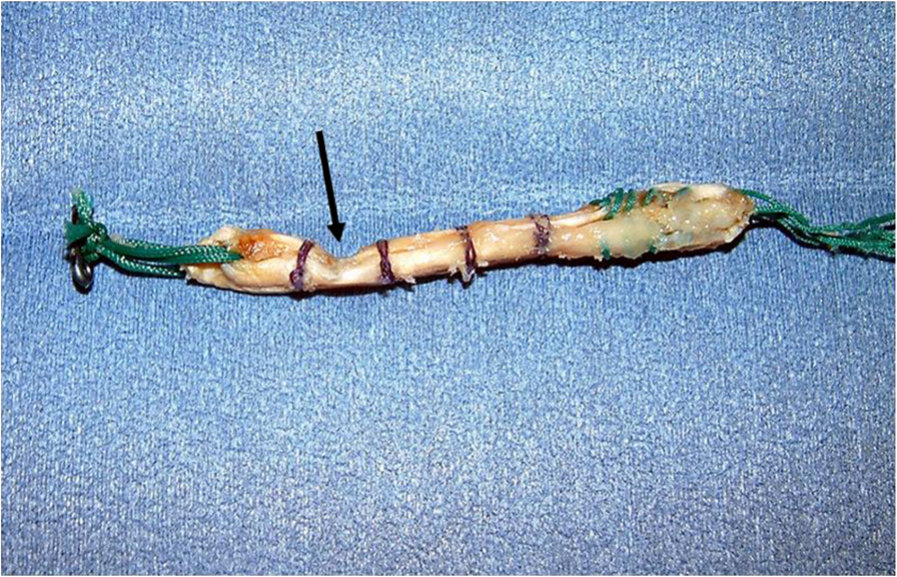
**Graft deformation after 500 cycles of 0 to 100-N anterior tibial loads.** Graft was deformed at the corner of the femoral tunnel aperture (arrow).

Neither graft pullout from the bone tunnel (fixation failure) nor graft rupture was found during cyclic loading tests. Although the tunnel was created to fit tightly with the graft diameter, gap formations or eccentric position of the graft within the tunnel were found in both SB and DB reconstruction after cyclic loading (Figure 6). Although there was no rupture or maceration of the graft, the graft was deformed at the corner of the femoral tunnel aperture (Figure 7). There was no macroscopic tunnel enlargement found.

## Discussions

The present study suggests that DB reconstruction is superior to SB reconstruction under cyclic load conditions. Repetitive sub-failure loads were applied to the human cadaveric knee and compared early stabilities between SB and DB reconstructed-knees by measuring the laxity increase. While cyclic loading test for ligament reconstruction have been usually performed to compare different types of graft and/or fixation device [24,26], this study compared the same type of graft and fixation device. DB reconstruction has several biomechanical and biological advantages, including reproduction of functionally different bundle, mimicking native ACL anatomy, increasing graft-tunnel contact area, and reducing stress at the graft-tunnel junction. After 500 cycles of 0 to 100-N, the laxity increase for DB reconstruction was significantly smaller when compared to SB reconstruction, confirming our hypothesis. This is another advantage of DB reconstruction.

The laxity increase consists of the graft slippage from the fixation device and permanent elongation of the graft construct including plastic deformation of the linkage materials and knot tightening [24,26,27]. Especially in the hamstring tendon, the larger graft elongation appeared to be caused by the longer running route of the hamstring construct due to the placement of the fixation devices, i.e. the Endobutton and the post screw outside of the bone tunnels [28,29]. The main cause of laxity increase is also considered to be due to graft slippage and graft elongation in both SB and DB reconstruction in this study. However, there was significant difference between them. After cyclic loading, the soft tissue graft becomes deformed within the tunnel as it goes the shortest route. A bigger tunnel in SB reconstruction might result in more eccentric positioning of the graft, and with more fixation loss. This may be one of the reasons why SB reconstruction had a larger laxity increase after cyclic loading.

**Figure 8:**
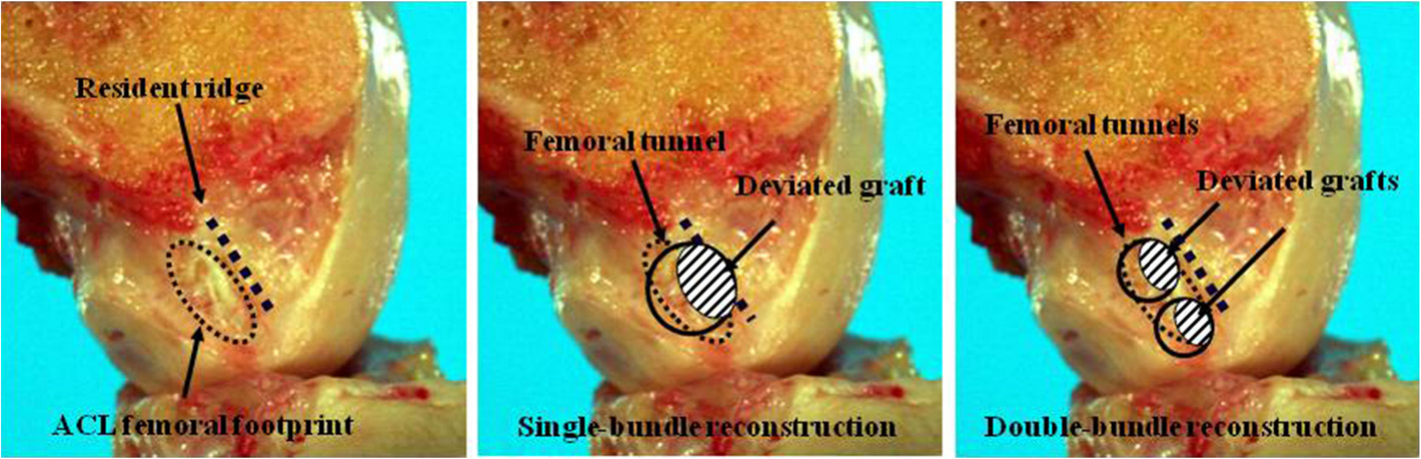
**Femoral footprint of ACL and resident ridge.** Although a double-bundle graft remains within the femoral footprint, a single-bundle graft might deviate into a non-anatomical position.

The goal of ACL reconstruction should be to closely restore the native ACL anatomy, which is considered to result in superior clinical outcomes [30]. Therefore, several anatomical studies have been conducted for anatomically accurate ACL reconstruction. Tibial insertion is located at a fossa in front of and lateral to the anterior tibial spine with oval shape [31]. On the other hand, femoral insertion is located at the medial aspect of the lateral femoral condyle just behind the “resident’s ridge” [32], in quite a narrow area (Figure 8-a). If this femoral footprint is mimicked, double-tunnels are better than single-tunnel [30]. Of course, a thicker single-graft can cover both the AM and PL bundle footprint. However, such single-graft may deviate into an eccentric position within the large bone tunnel as shown in this study (Figure 8-b). This results in not only stability deterioration but also non-anatomical positioning of the graft. Graft deviations also occur in DB reconstruction, however they are smaller within smaller double tunnels than that in SB reconstruction and grafts are still located within the ACL footprint (Figure 8-c).

Recently the hamstring tendon has become the preferred graft substitute by surgeons who are concerned that harvesting the bone-patellar tendon-bone (BTB) graft may impair the function of the knee extensor mechanism, and hence perform DB reconstruction. Although the hamstring tendon has several advantages making it superior to a BTB graft, it also has several disadvantages. The tendon fixation to the bone is still a challenge in spite of the numerous surgical devices available [33]. Relatively slow maturation of the tendon-bone interface accentuates the need for retaining the graft firmly when the reconstructed-knee is subject to a repetitive load [34]. In addition, graft deformation within the tunnel is another disadvantage of this graft. On the other hand, the bone plug of the BTB graft may prevent such deviation within the tunnel. This may be one of the reasons why stability after ACL reconstruction with a BTB graft is better than that of the hamstring tendon [35].

There were several limitations in this study. The first was the use of the same knee specimens repeatedly for SB and DB procedures. It is very difficult to obtain fresh cadaver knees in our country, requiring the need to reuse some specimens. Although the reconstructions were performed in random order, mechanical deterioration of the bone might influence results of the second reconstruction. To decrease influence from the first reconstruction, bone plugs packed into the first tunnel were harvested from the femoral condyle of other specimens which had good bone quality. Another limitation was that the average age of the specimens was relatively high. Reduced bone quality of older human subjects predisposes to graft slippage from the fixation device placed within the bone tunnel [36,37]. Although our testing devices permitted 4-DOF, only AP displacements at 30° of knee flexion were measured. This is a limitation compared to recent advances in technology, such as a robotic manipulator, which can assess 3-dimensional motion at any knee flexion angle. However, we believe that the materials testing machine is more suitable for cyclic loading test because it takes a long time. Thirty degrees of knee flexion was chosen for this biomechanical study, because it is the most critical angle in ACL and tensions of the AM and PL bundle are considered to be equal based on previous biomechanical studies. If another angle was chosen for the cyclic loading test, uneven distribution of forces on the AM and PL bundle would have occurred in DB reconstruction [4,15].

Regardless of these limitations, this study clearly showed a different advantage of DB reconstruction. If we use a soft-tissue graft, such as a hamstring tendon, the graft deviates within the tunnel after cyclic loading to take the path of shortest distance. This might deteriorate knee stability after early aggressive rehabilitation [17-21]. Furthermore, a thicker SB graft may end up in a non-anatomical position contrary to the surgeon’s intention. We are not stating that SB is inadequate for ACL reconstruction, however, surgeons should know the material properties of each graft and choose the optimal graft substitute for each reconstruction technique.

## Conclusions

Although ATT and “laxity increase” was significantly increased in both SB and DB reconstruction with hamstring tendon after cyclic load testing, there was significant difference between them. DB reconstruction might be superior to SB reconstruction in preventing increase in laxity under cyclic loading.

## Competing interests

The authors declare that they have no competing interests.

## Authors’ contributions

SN mainly participated in data collection, and drafted the manuscript. YI conceived the main idea, participated in the design of the study, and its revision and coordination. ET participated in the development of the study question and in data collection. YY and HT participated in data collection, and conducted statistical analyses. ST participated in the revision of the manuscript. All authors read and approved the final manuscript.
